# Unsweetened Natural Cocoa Powder Has the Potential to Attenuate High Dose Artemether-Lumefantrine-Induced Hepatotoxicity in Non-Malarious Guinea Pigs

**DOI:** 10.1155/2016/7387286

**Published:** 2016-07-14

**Authors:** Isaac Julius Asiedu-Gyekye, Kennedy Kwami Edem Kukuia, Abdulai Mahmood Seidu, Charles Antwi-Boasiako, Benoit Banga N'guessan, Samuel Frimpong-Manso, Samuel Adjei, Jonathan Zobi, Abraham Terkpertey Tettey, Alexander Kwadwo Nyarko

**Affiliations:** ^1^Department of Pharmacology and Toxicology, University of Ghana School of Pharmacy, College of Health Sciences, Legon, Ghana; ^2^Department of Chemical Pathology, School of Biomedical and Allied Health Sciences, College of Health Sciences, Korle-Bu, Ghana; ^3^Department of Physiology, School of Biomedical and Allied Health Sciences, College of Health Sciences, Korle-Bu, Ghana; ^4^Department of Pharmaceutical Chemistry, University of Ghana School of Pharmacy, College of Health Sciences, Legon, Ghana; ^5^Department of Animal Experimentation, Noguchi Memorial Institute for Medical Research, College of Health Sciences, Accra, Ghana

## Abstract

*Objective*. This study investigated the elemental composition of unsweetened natural cocoa powder (UNCP), its effect on nitric oxide, and its hepatoprotective potential during simultaneous administration with high-dose artemether/lumefantrine (A/L).* Method*. Macro- and microelements in UNCP were analyzed with EDXRF spectroscopy. Thirty (30) male guinea-pigs were then divided into five groups. For groups 3 (low-dose), 4 (medium-dose), and 5 (high-dose), the animals received oral UNCP prophylactically for 14 days. Group 1 received distilled water (14 days) and group 2 A/L for the last 3 days (days 12 to 14). After euthanisation, biochemical and histopathological examinations were carried out in all groups.* Results*. Phytochemical analysis of UNCP showed the presence of saponins, flavonoids, tannins, and cardiac glycosides. Thirty-eight (38) macro- and microelements were found. UNCP produced significant decreases in ALT, ALP, GGT, and AST levels. A significant increase in total protein levels was observed during A/L+UNCP administration in comparison to 75 mg/kg A/L group. Histopathological examinations buttressed the protective effects of cocoa administration. UNCP administration increased nitric oxide levels 149.71% (*P* < 0.05) compared to controls.* Conclusion*. UNCP increases nitric oxide levels and has hepatoprotective potential during A/L administration. A high level of copper was observed which may be detrimental during high daily consumptions of UNCP.

## 1. Introduction 

Malaria is an infection transmitted by the female anopheles mosquito. It is a major public health issue in the tropics and one of the world's leading infectious killer diseases. The high death rate resulting from malaria cannot be overemphasised especially in some parts of Africa. In Ghana 3.5 million people contract malaria every year [1, 2]. Resistance is a major setback in the management of malaria and has therefore necessitated countries to review and implement new antimalarial drug policies to ensure effective case management to reduce both morbidity and mortality [3]. Due to increased therapeutic efficacy, decreased cytotoxicity, and delay or prevention of the development of drug resistance, combination drug regimens is recommended over monotherapy [4]. Artemisinin-based combination therapy (ACT) has been recommended for use by the WHO [5, 6]. Artemether/lumefantrine (A/L) is one of the combination therapies currently used.

Artemisinin derivatives have impressive parasiticidal properties* in vivo* and* in vitro* but currently there have been issues of treatment failures, resistance, and increasing cases of hepatotoxic effects [7–9]. Some countries have considered increasing the dose of the A/L in treatment in order to arrest the issue of resistance [10] but increase in dose implies that there will be increased side effects, adverse reactions, and hepatotoxic effects [9]. In fact, there are concerns about frequent usage of A/L on some organ systems [9]. Considering the fact that so far A/L is one of the most effective combination therapies, the issue of drug-induced hepatotoxicity needs to be addressed. Another effect of A/L is its effect on nitric oxide levels, where it has been found to reduce nitric oxide levels. However, other studies show that A-L increase nitric oxide levels as a compensatory mechanism in cases of reduced nitric oxide levels [9].

Unsweetened natural Cocoa powder (UNCP) is used as a beverage and nutraceutical amongst Ghanaians. The antioxidant properties of cocoa powder have been well studied [11–13] and even found to be unchanged after various manufacturing processes [14]. It contains antioxidant polyphenols called flavonols reported to have hepatoprotective [15, 16] and antimalarial effects [17, 18]. The chemical composition of cocoa has been well investigated using various methods. UNCP contains about 1.9% theobromine and 0.21% caffeine [19–21]. Polyphenols in various compounds have also been proven in several studies to exert hepatoprotective activity [22–25]. For example, polyphenol-rich fractions prepared from walnut kernel pellicles have been assessed for its hepatoprotective effect in mice [23]. Studies conducted on cocoa powder has shown its quantitative components such as 14 N-phenylpropenoyl-L-amino acids, N-[4′-hydroxy-(E)-cinnamoyl]-L-tryptophan, and N-[4′-hydroxy-3′-methoxy-(E)-cinnamoyl]-L-tyrosine [26]. Other studies have also proven the roles of polyphenols such as cynarin, isochlorogenic acid, chlorogenic acid, luteolin-7-glucoside, and two organic acids, caffeic and quinic, from Cynara scolymus in the hepatoprotective activity [27]. Besides, cocoa or flavonols increase nitric oxide levels which have also been found to have hepatoprotective effects in acute liver injury by virtue of their antioxidant properties [18, 28, 29]. Further, the tannin, glycoside, flavonoid, and saponins have also been implicated in their hepatoprotective effect in several studies. The total flavonoid content of UNCP has also been determined [30–34]. This is a strong background to investigate the beneficial effect UNCP might have in ameliorating the hepatotoxic effect of this important drug A/L.

It is important in spite of all these component-based effects of UNCP to note that both micro- and macroelements present in UNCP are also capable of interfering with the availability of secondary metabolites in UNCP which may easily modulate their pharmacological effects [35]. The presence of toxic heavy metals in medicinal plants can on the other hand pose a threat to the health of consumers [35, 36]. Though UNCP contains some elemental particles, correlation between the pharmacological activity of UNCP and these macro- and microelements has not been established. It has been established, for example, that zinc has beneficial hepatoprotective action [37, 38]. Simultaneous ingestion of UNCP and A/L is a common practice amongst Ghanaians suffering from uncomplicated malaria undergoing treatment with A/L. It is important to establish the beneficial effect and visible adverse effect of the simultaneous consumption of UNCP and A/L which is a way of life for Ghanaians especially with the affordable price of UNCP and highly subsidised A/L.

This study seeks to determine the elemental composition of UNCP and its effect on nitric oxide levels and to assess its hepatoprotective potential against A/L-induced liver toxicity during their simultaneous ingestion in male guinea pigs.

## 2. Materials and Methods

### 2.1. Energy Dispersive X-Ray (EDXRF) Measurements

Sample of UNCP (Batch number BT620IT; FDA/DK06-070) was acquired from a supermarket. The sample was sieved using sieve of 180 microns and three samples prepared and sieved with a mesh size (aperture) of 180 *μ*m into fine powder. This was kept in dry well-labelled containers. Before pelletation, the sample was kept in an oven at 60°C overnight. Triplicate weighed samples, 4000 mg/sample, were added separately to 900 mg Fluxana H Elektronik BM-0002-1 (Licowax C micropowder PM-Hoechstwax) as binder (due to their morphology and the loose nature); the mixture was homogenized using the RETSCH Mixer Mill (MM301) for 3 min and pressed manually with SPECAC hydraulic press for 2 min with a maximum pressure limit of 15 tons (15000 kg) into pellets of 32 mm in diameter and 3 mm thickness for subsequent XRF measurements. Time between pelletation and measurement was kept short to avoid deformation of the flat surfaces of the pellets [39]. A factory calibrated Spectro X-Lab 2000 spectrometer (from the Geological Survey Department, Accra, Ghana) enhanced with three-axial geometry was used for the simultaneous analysis and measurement of the elemental content of the samples.

### 2.2. Preparation of UNCP Solution

Calculated amount (9.6 g) of UNCP was dissolved in warm distilled water (40 mL) with stirring (till everything went into solution) making a concentration of 240 mg/mL (of the UNCP). This was administered to the guinea pigs in groups 3, 4, and 5 at their respective doses via oral gavage.

### 2.3. Preparation of A/L Solution

A concentration of 20 mg/mL of dispersible A/L (with reference to artemether) was prepared and administered to the guinea pigs in the UNCP treated groups at a dose of 75 mg/kg body weight daily for 3 days via oral gavage. Dosage was calculated with reference to the dose of artemether in the drug combination. To achieve this, seventy (70) tablets of Novartis Coartem® dispersible tablets (20/120 mg), which are equivalent to 1400 mg of artemether, were dissolved in 70 mL of distilled water and stirred until completely homogenous.

In all cases, fresh solutions of UNCP and A/L were prepared before each dosing.

### 2.4. Phytochemical Analysis

Phytochemical analysis was conducted to determine the various constituents in the unsweetened natural cocoa [40].

#### 2.4.1. Saponin Test

About 0.5 g of UNCP was added to water in a test tube. The test tube was shaken to observe foam formation.

#### 2.4.2. Tannins Test

About 0.5 g of UNCP was dissolved in 80% of aqueous methanol (10 cm^3^). Freshly prepared iron (III) chloride solution was added and observations were made on colour changes.

#### 2.4.3. Flavonoids Test

About 0.1 g of UNCP was added to 80% ethanol (15 cm^3^). Magnesium turnings were added to the filtrate followed by concentrated HCl (0.5 cm^3^) and observed for colour changes within 10 minutes.

#### 2.4.4. Cardiac Glycoside Test

About 0.5 g of UNCP was dissolved in chloroform (2 cm^3^) in a test tube after which concentrated sulphuric acid was carefully added down the side of the test tube to form a lower layer.

### 2.5. Animal Husbandry

Thirty (30) male guinea pigs (450 g and 600 g) were purchased from the Noguchi Memorial Institute for Medical Research, University of Ghana, for this experiment. The animals were acclimatized to the laboratory environment for one week before being used in the study and were provided with Sankofa pellet feeds and tap water* ad libitum*. The room temperature was maintained at 20–23°C with 12 : 12 hour light/dark cycle. Spontaneous behaviours of all guinea pigs were observed in cages before experimental procedures were carried out. No animals showed signs of illness before the experimental phase. The study protocol was approved by the departmental ethical and protocol review committee and the Noguchi Memorial Institute for Medical Research Institutional Animal Care and Use Committee with protocol approval number 2013-01-3E.

### 2.6. Drug Administration

The guinea pigs were grouped into five, with groups 3 to 5 receiving the UNCP at 300, 900, and 1500 mg/kg, respectively, for 14 days. Doses of A/L were administered for the last 3 days of cocoa administration. Group 2 animals were given only A/L for the last 3 days whereas group 1 received vehicle (water) only. The weights of the animals were taken weekly and the doses administered adjusted accordingly. All experiments carried out on animals conformed to the guidelines on ethical standards for inducing toxicity in animals (NLC, 1996).

 
*Group 1*. Control (distilled water only)
 
*Group 2*. 75 mg/kg A/L (last 3 days)
 
*Group 3*. Cocoa 300 mg/kg (14 days) + 75 mg/kg A/L (last 3 days)
 
*Group 4*. Cocoa 900 mg/kg (14 days) + 75 mg/kg A/L (last 3 days)
 
*Group 5*. Cocoa 1500 mg/kg (14 days) + 75 mg/kg A/L (last 3 days).

### 2.7. Biochemical Assays

Blood samples were collected from the descending aorta and aliquoted into EDTA-2K tubes and plain tubes, respectively, at the end of the dosing period. This was done after euthanisation of the animals under ether anaesthesia. The EDTA blood was immediately analyzed for haematological parameters using the SYSMEX Haematology Autoanalyser (Kobe, Japan) while sera prepared from blood in plain tubes were used for biochemical examinations including clinical chemistry measurements such as alanine aminotransferase (ALT) or glutamic pyruvic transaminase (SGPT) levels, alkaline phosphatase (ALP), Serum Glutamic Oxaloacetic Transaminase (SGOT) or aspartate transaminase, and Gamma Glutamyl Transpeptidase (GGT). These were measured as liver function tests (LFT) to give an indication of the state of the liver.

Nitric oxide levels were also measured using the Griess Reagent System. The total nitric oxide kit by R&D Systems was used in this study. In this system, nitrate is converted to nitrite using nitrate reductase after which the total nitrite is measured.

The principle of this assay determines nitric oxide concentration based on the enzymatic conversion of nitrate to nitrite by nitrate reductase. The reaction is followed by colorimetric detection of nitrite as an azo dye product of the Griess Reaction. The Griess Reaction is then based on the two-step diazotization reaction in which acidified NO_2_^−^ produces a nitrosating agent, which reacts with sulfanilic acid to produce the diazonium ion. This ion is then coupled to* N*-(1-naphthyl) ethylenediamine to form the chromophoric azo-derivative which absorbs light at 540–570 nm.

### 2.8. Histopathological Studies

Guinea pigs were euthanized and their livers were swiftly excised and washed with 0.9% saline. The livers were stored in 10% neutral buffered formaldehyde. The liver tissues were then cut and sectioned. A microtone was used to cut 2 *μ*m thick liver slices and stained with haematoxylin-eosin for examination. The stained tissues were observed with an Olympus microscope (BX-51) and photographed by INFINITY 4 USB Scientific Camera (Lumenera Corporation, Otawa, Canada).

The study protocol was approved by the departmental ethical and protocol review committee and the Noguchi Memorial Institute for Medical Research Institutional Animal Care and Use Committee with protocol approval number 2013-01-3E.

### 2.9. Statistics

The results are reported as mean ± SEM. Statistical analysis was performed using one-way analysis of variance (ANOVA) followed by student* Newman*-*Keuls post hoc* test. Statistical significance was set at *P* < 0.05; Dunnet Multiple Comparison Test was used in the analysis of the nitric oxide levels. All statistical analyses were performed using Graph Pad prism 5 software.

## 3. Results

### 3.1. Energy Dispersive X-Ray (EDXRF) Measurements

A total of thirty-eight (38) elements comprising 12 macroelements, (sodium (Na), magnesium (Mg), aluminium (Al), silicon (Si), phosphorus (P), sulphur (S), chlorine (Cl), potassium (K), calcium (Ca), titanium (Ti), manganese (Mn), and iron (Fe), and 26 microelements, vanadium (V), chromium (Cr), cobalt (Co), nickel (Ni), copper (Cu), zinc (Zn), gallium (Ga), arsenic (As), rubidium (Rb), strontium (Sr), yttrium (Y), zirconium (Zr), niobium (Nb), molybdenum (Mo), antimony (Sb), iodine (I), cesium (Cs), barium (Ba), lanthanum (La), cerium (Ce), hafnium (Hf), tantalum (Ta), lead (Pb), bismuth (Bi), thorium (Th), and uranium (U)) (Table 1), were identified and evaluated.

### 3.2. Phytochemical Analysis

Phytochemical analysis of unsweetened natural cocoa powder showed the presence of saponins, flavonoids, tannins, and cardiac glycosides.

### 3.3. Biochemical Assays

#### 3.3.1. Liver Function Tests

**Figure 1:**
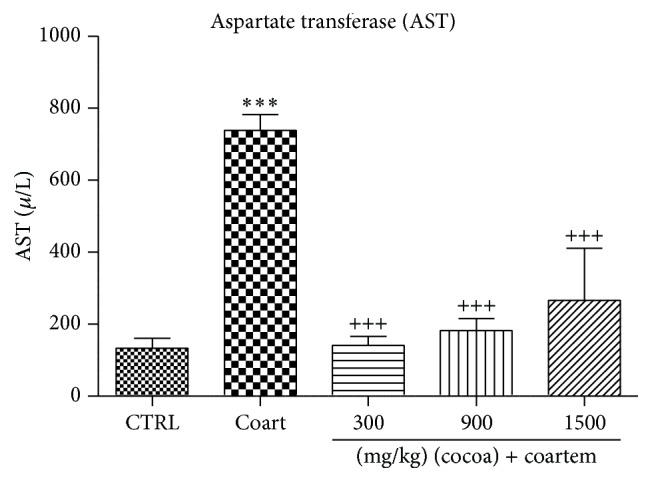
Changes in AST levels during a 14-day administration of UNCP in male guinea pigs followed by a 3-day A/L administration. Values are expressed as mean ± SEM, *n* = 6. The differences among the mean were analyzed using one-way ANOVA followed by* Newman-Keuls post hoc* analysis. ^*∗∗∗*^ means *P* < 0.0001 when compared to the control (distilled water) and ^+++^ means *P* < 0.0001 when compared to the A/L group.

AST levels increased in animals that received A/L 75 mg/kg by 81.97% when compared to the group that received distilled water (control group) while those in the UNCP administered group decreased with percentage change of 80.91%, 75.33%, and 63.86%, respectively, when compared with the A/L administered group (*P* < 0.05) (Figure 1).

**Figure 2 fig2:**
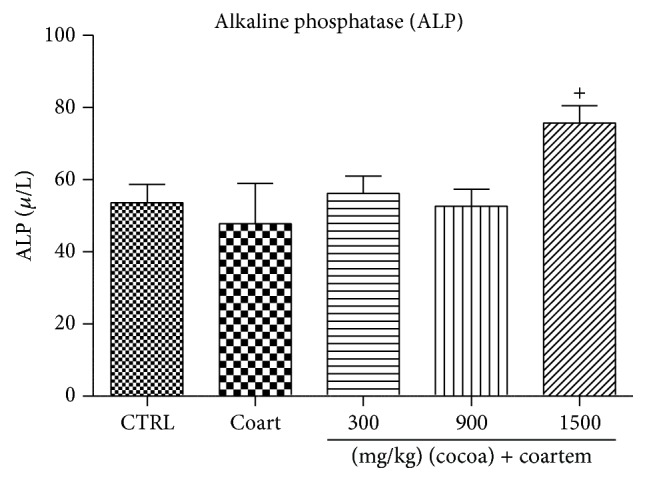
Changes in ALP levels during a 14-day administration of UNCP in male guinea pigs followed by a 3-day A/L administration. Values are expressed as mean ± SEM, *n* = 6. The differences among the mean were analyzed using one-way ANOVA followed by* Newman-Keuls post hoc* analysis. ^+^ means *P* < 0.05 when compared with A/L group.

ALP levels decreased in group 2 by 10.82% when compared to group 1. ALP levels in groups 3, 4, and 5 increased with percentage change of 14.95%, 9.13%, and 36.94%, respectively, when compared with group 2 (*P* < 0.05) (Figure 2).

**Figure 3 fig3:**
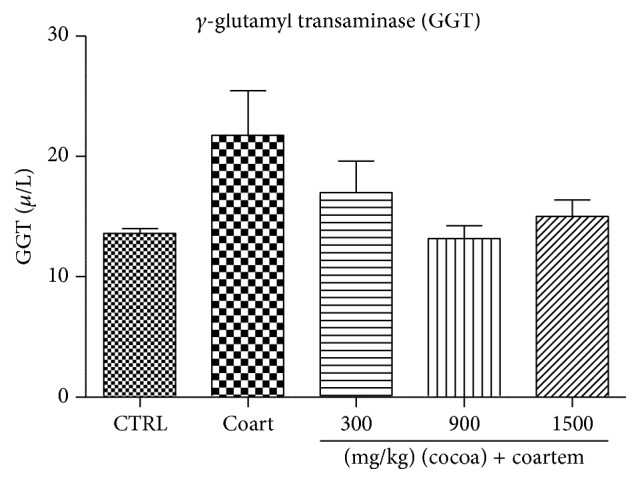
Changes in GGT levels during a 14-day administration of UNCP in male guinea pigs followed by a 3-day A/L administration. Values are expressed as mean ± SEM, *n* = 6. The differences among the mean were analyzed using one-way ANOVA followed by* Newman-Keuls post hoc* analysis.

Levels of GGT increased in animals that received 75 mg/kg A/L by 37.61% and a decrease by 39.5% in the cocoa treated group when compared to the control group (*P* > 0.05) (Figure 3).

**Figure 4:**
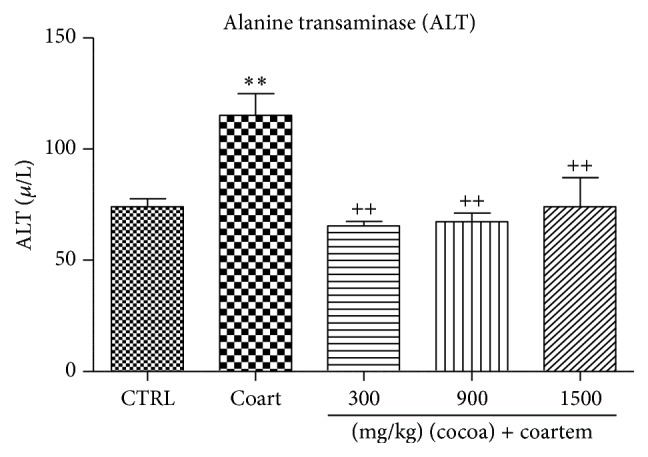
Changes in ALT levels during a 14-day administration of UNCP in male guinea pigs followed by a 3-day A/L administration. Values are expressed as mean ± SEM, *n* = 6. The differences among the mean were analyzed using one-way ANOVA followed by* Newman-Keuls post hoc* analysis. ^*∗∗*^ means *P* < 0.001 when compared to the control (distilled water) and ^++^ means *P* < 0.001 when compared to the A/L group.

ALT levels increased in the group dosed at 75 mg/kg A/L by 35.76% when compared to the control group. ALT levels at doses of 300 mg/kg, 900 mg/kg, and 1500 mg/kg decreased with percentage change of 43.05%, 41.49%, and 35.76%, respectively, when compared to the A/L administered group (*P* < 0.05) (Figure 4).

**Figure 5:**
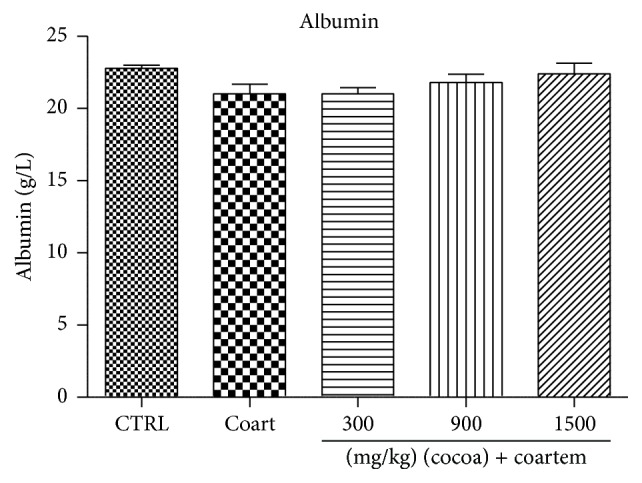
Changes in ALP levels during a 14-day administration of UNCP in male guinea pigs followed by a 3-day A/L administration. Values are expressed as mean ± SEM, *n* = 6. The differences among the mean were analyzed using one-way ANOVA followed by* Newman-Keuls post hoc* analysis.

**Figure 6:**
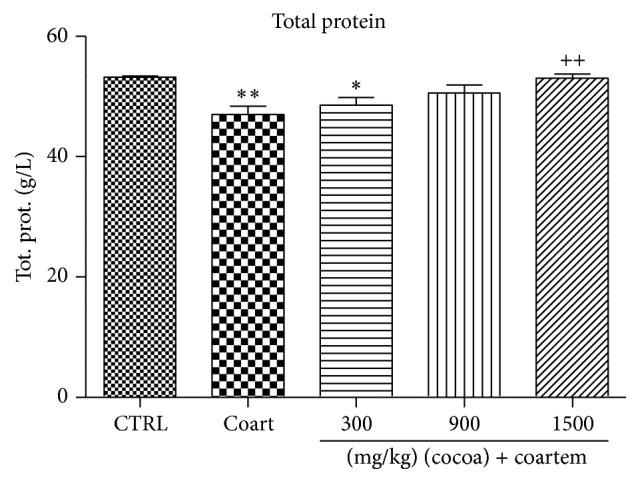
Changes in total protein levels during a 14-day administration of UNCP in male guinea pigs followed by a 3-day A/L administration. Values are expressed as mean ± SEM, *n* = 6. The differences among the mean were analyzed using one-way ANOVA followed by* Newman-Keuls post hoc* analysis. ^*∗*^ means *P* < 0.05, ^*∗∗*^ means *P* < 0.001 when compared with control (distilled water), and ^++^ means *P* < 0.001 when compared with the A/L group.

**Figure 7:**
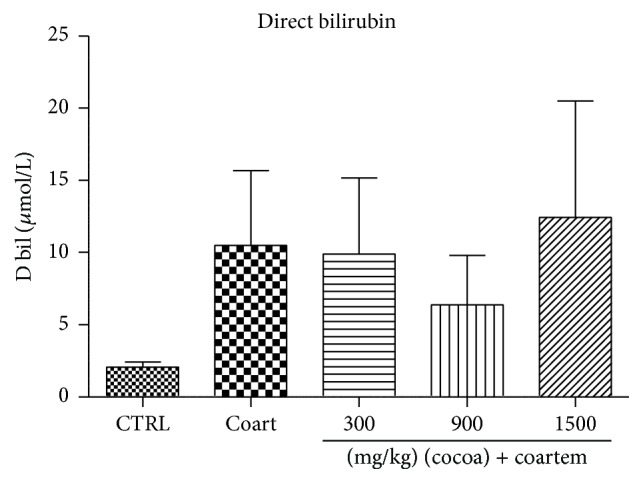
Changes in direct bilirubin levels during a 14-day administration of UNCP in male guinea pigs followed by a 3-day A/L administration. Values are expressed as mean ± SEM, *n* = 6. The differences among the mean were analyzed using one-way ANOVA followed by* Newman-Keuls post hoc* analysis.

Serum albumin levels reduced slightly in the A/L administered group while the levels slightly increased in the animals treated with UNCP compared to the controls (*P* > 0.05). Total protein levels decreased in the A/L group by 7.89% (Figure 5) while bilirubin levels increased by 80.4% when compared to the control groups (Figure 7). In the UNCP administered group, total protein levels increased up to 12.77% in the 1500 mg/kg group while the bilirubin levels reduced up to 39.35% in the 900 mg/kg group, (*P* > 0.05) (Figure 6).

#### 3.3.2. Nitric Oxide Levels

Group 3 produced the greatest increase (147.33 ± 117.78, *P* < 0.05, i.e., 149.71%) in nitric oxide followed by group 4 (79.21 ± 36.24, *P* < 0.05, i.e., 34.25%) and then group 5 (61.88 ± 3.83, *P* < 0.05, i.e., 4.88%) when compared to group 1 (Figure 9). At a dosage of 900 mg/kg (cocoa only), considered to be the optimal in most studies, there was just a slight increase in nitric oxide (36.92 ± 3.65, *P* = 00.0024).

#### 3.3.3. Histopathological Analysis

The histological examination of liver samples was based on changes associated with exposure of liver tissues to toxins. This is mainly inflammatory associated changes ranging from acute to chronic changes such as dilation of central and microcirculatory vessels, congestion of the central vein, leucocyte infiltration, and disturbances in the liver parenchyma (necrosis). Sections of liver from guinea pigs that received only a 3-day HD A/L administration (75 mg/kg) revealed disturbed liver parenchyma (necrotised liver cells) with highly congested and dilated central vein as well as lymphocyte infiltration (Figure 10(a)). This finding is indicative of a severe liver damage (LDS of 4-5) [41].

In contrast, examination of liver sections from 14-day unsweetened natural cocoa administration (300, 900, and 1500 mg/kg) followed by a 3-day A/L administration (75 mg/kg) showed undisturbed liver parenchyma, uncongested but dilated central vein, and slightly dilated sinusoids (Figures 10(c), 10(d), and 10(e)). This is indicative of a mild change (LDS of 1-2) with a high degree of reversibility. Liver sections from the control group that received only distilled water showed undisturbed liver parenchyma with uncongested central veins (Figure 10(b)).

## 4. Discussion

The above study has shown that UNCP contains 38 elemental particles comprising 12 macro- and 26 microelements believed to play roles in executing pharmacological effect of natural products. The phytochemical constituents of the powder were also identified as flavonoids, tannins, saponins, terpenoids, and glycosides.

Hepatocyte membrane distortion is associated with membrane leakage of the hepatocyte cytosolic contents which manifests by significant elevation of serum or plasma enzymes. ALT, AST, and ALP have been shown to be reliable markers of acute hepatocellular damage. Among the marker enzymes, ALT is the most reliable because AST is known to be abundant in the cardiac muscles, skeletal muscles, kidneys, and testes. Thus, any disease state affecting hepatic tissues significantly elevates the serum level of these enzymes [42, 43].

In our study, A/L increased the levels of ALT, AST, GGT, and bilirubin, while the levels of albumin and total protein were reduced indicating the presence of hepatotoxicity (Figures 1 [Fig fig2] [Fig fig3]–4). The increases in AST and ALT were dose dependent. Normally, hepatotoxicity is accompanied by a significant rise in ALT levels more than three times the upper limit of normal. ALP levels also increase more than twice the upper limit level or total bilirubin more than twice when associated with increased ALP or ALT. Further, liver damage could be either hepatocellular (predominately initial alanine transferase elevation) or cholestatic (initial alkaline phosphatase rise) types. However, they are no mutually exclusive and mixed types of injuries that are often encountered [44]. In order to differentiate liver diseases from elevated ALP related conditions, serum GGT measurement was conducted. Elevations in GGT may indicate that the integrity of the hepatocyte membranes has been compromised [44, 45].

A/L administration was accompanied by a high elevation of GGT levels. Similar studies have also found A/L and other artemisinin derivatives to have hepatotoxic effects [9, 45–47]. Besides, other antimalarial drugs such as chloroquine, amodiaquine, quinine, and halofantrine have also been reported to elevate serum ALT and ALP and may induce hepatic damage [9, 46, 48–50]. It has also been shown in other studies that A/L increases the level of oxidants such as superoxides (O^2−^) and peroxides (H_2_O_2_) which leads to oxidative stress [48]. Though this was not measured in this study, it is known that reactive oxygen species (ROS) generated during the process of drug biotransformation can bind and react with cellular components in the liver to cause hepatic injury, thus impairing liver function [49]. Taking these mechanisms into consideration, it is plausible that drugs that have antioxidant activity or have the ability to reduce oxidative stress can be useful in preventing the deleterious effects of A/L on the liver. It is noteworthy that the elevations in serum liver enzymes were absent in guinea pigs pretreated with UNCP for 14 days before A/L administration. UNCP and its derived products have been shown to contain important antioxidant polyphenols that inhibit different tumoral processes and exhibit antioxidant and anti-inflammatory properties [50–53]. The hepatoprotective effect exhibited by cocoa may likely be due to the antioxidative effects of their polyphenols [54–56].

**Figure 8:**
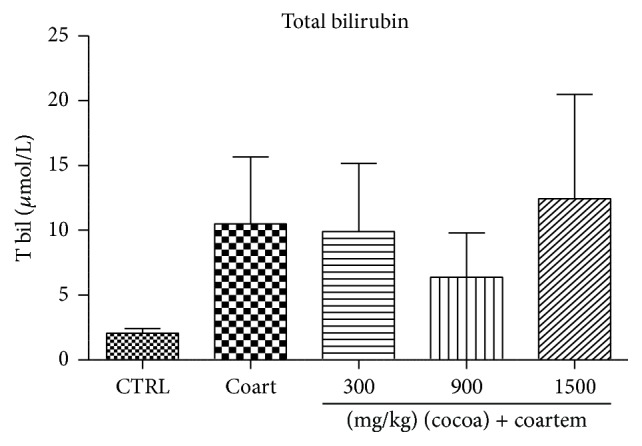
Changes in total bilirubin levels during a 14-day administration of UNCP in male guinea pigs followed by a 3-day A/L administration. Values are expressed as mean ± SEM, *n* = 6. The differences among the mean were analyzed using one-way ANOVA followed by* Newman-Keuls post hoc* analysis.

Furthermore, UNCP increased total proteins and albumin levels in the animals unlike the A/L administered group (Figures 5 and 6). Total protein and albumins are used to assess the synthetic functions of the liver. The diminution of total protein and albumin levels further support the hepatotoxic effects of A/L. In contrast, UNCP induced significant elevation in total proteins, which may be a reflection of its hepatoprotective effect. UNCP did not cause significant increase in albumin. Also, studies indicate that cocoa does not significantly increase albumin levels [57]. This buttresses the finding from this experiment. Bilirubin levels were not significantly affected by both A/L and UNCP (Figure 8).

Hepatoprotective effect of UNCP was further corroborated by the fact that there were no histological abnormalities following the administration of UNCP before A/L administration. Our histopathological studies showed damaged liver tissues in animals that received A/L alone evidenced by disturbed (necrotic) liver parenchyma (NeLP), a highly congested and dilated central vein (CCV) and lymphocytic infiltration (LYM) in all animals (Figure 10(b)), a situation that could be described as severe with a LDS between 4 and 5 according to Krastev [41]. UNCP administration reduced the extent of liver damage evidenced by the undisturbed liver parenchyma with an uncongested but dilated central vein (mild liver damage). The total protein levels tend to be restored (Figure 6).

Animals that received various doses (300 mg/kg, 900 mg/kg, and 1500 mg/kg) of UNCP showed a normal uncongested and dilated central vein. However, in one of the animals that received 1500 mg/kg UNCP, there was observed, dilated, and congested central vein. High levels of copper in the blood have been shown to be responsible for liver and gastrointestinal disorders [58]. Thus, this observation of the compromised liver integrity might probably be due to the high levels of copper (Table 1) at this high dose of UNCP. This level of copper has also been observed in our study where energy dispersive X-ray (EDXRF) analyses of UNCP showed the presence of both macro- and microelements including copper 0.2984 mg ± 1.71 per 4 g of UNCP (Table 1). The copper content of 1500 mg/kg UNCP exceeds the normal recommended daily allowance (RDA) of 900 *μ*g (i.e., more than 103.6% of RDA). Thus, caution should be taken against frequent high consumption of UNCP as a beverage.

This study reports for the first time the hepatoprotective effect of UNCP against A/L-induced hepatic damage in guinea pigs. Similar effect of UNCP has been demonstrated in other studies where it was shown that UNCP reduced liver damage in mice infected with* Plasmodium berghei* [59], prevented alcohol-induced hepatic damage in rats [60], and protected against liver and renal damage by carbon tetrachloride [61].

**Figure 9:**
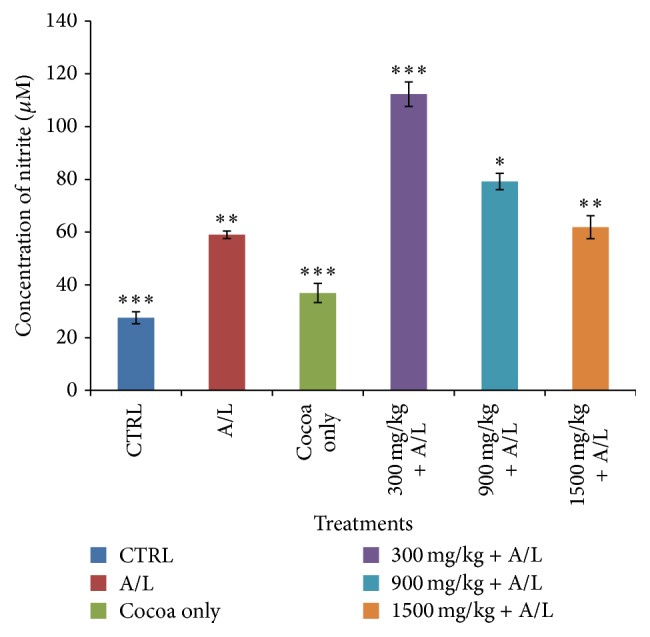
Effect of UNCP (LD = 300, MD = 900, and HD = 1500 mg/kg) on nitrite concentrations in plasma of guinea pigs during A/L administration. Values are mean ± SD (*n* = 5) and ^*∗*^ *P* < 0.05, ^*∗∗*^ *P* < 0.01, and ^*∗∗∗*^ *P* < 0.001 compare to the control (one-way ANOVA followed by a* Dunnett's* multiple comparison test). The low dose UNCP (300 mg/kg) + A/L produced the greatest increase (147.33 ± 117.78, *P* < 0.05, i.e., 149.71%) in nitric oxide followed by medium dose UNCP + A/L (79.21 ± 36.24, *P* < 0.05, i.e., 34.25%) and then high dose UNCP + A/L (61.88 ± 3.83, *P* < 0.05, i.e., 4.88%) when compared to the A/L only group.

**Figure 10:**
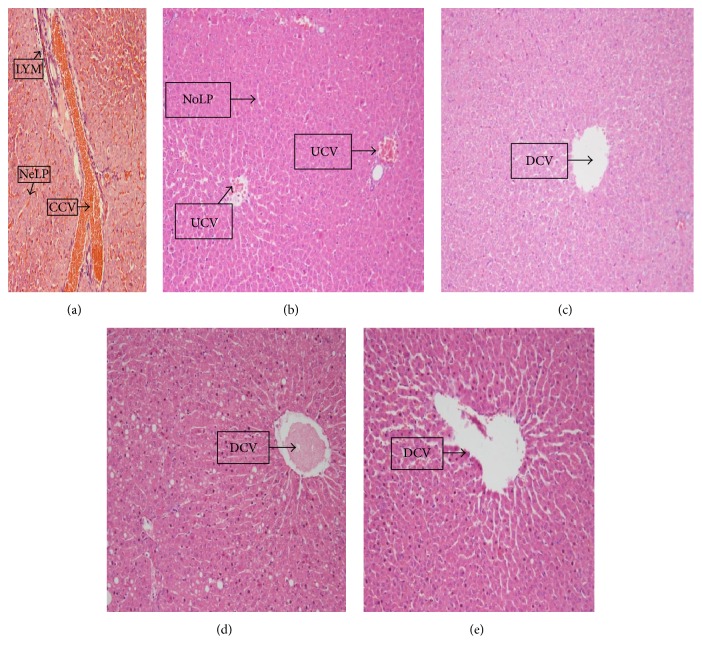
A representation of sections of liver from guinea pigs that received (a) only a 3-day HD A/L administration (75 mg/kg/bwt) showing disturbed (necrotic) liver parenchyma (NeLP), a highly congested and dilated central vein (CCV), and lymphocytic infiltration (LYM). (b) Distilled water (control) showing undisturbed liver parenchyma (NoLP) with uncongested central veins (UCV). Note the regularity of liver cell plates, microcirculatory zones, and sinusoids. (c) A 14-day UNCP administration (300 mg/kg/bwt) followed by a 3-day A/L administration (75 mg/kg/bwt) showing undisturbed liver parenchyma with an uncongested but dilated central vein (DCV). (d) A 14-day UNCP administration (900 mg/kg/bwt) followed by a 3-day A/L administration (75 mg/kg/bwt) showing undisturbed liver parenchyma with an uncongested but dilated central vein (DCV). (e) A 14-day UNCP administration (1500 mg/kg/bwt) followed by a 3-day A/L administration (75 mg/kg/bwt) showing undisturbed liver parenchyma with an uncongested but dilated central vein (DCV) (H & E stain ×40).

The effect of UNCP on nitric oxide was also investigated. Nitric oxide has been found to have hepatoprotective effects in acute liver injury. The 900 mg/kg UNCP administered group showed an increase in nitric oxide levels. This dose level has been identified by other researchers as the optimum dose for beneficial effects of cocoa. The observed moderate nitric oxide increases that are beneficial [30] could be attributed to the flavonoid content of the unsweetened natural cocoa [29]. Cocoa increases nitric oxide levels in humans as observed for studies involving consumption for flavonoid rich chocolate and cocoa drinks [62, 63]. Superoxides and peroxides have been found to scavenge nitric oxide [61]. The rise in nitric oxide caused by A/L observed in the present study may be consistent with others who have suggested that to be as a result of a compensatory mechanism trying to restore the nitric oxide level [62]. Nitric oxide increased in the UNCP and A/L combinations with the greatest increments in animals receiving 300 mg/kg and 900 mg/kg UNCP. This may indicate that the hepatoprotective effect is likely to be more pronounced in the animals in these groups as confirmed by both the biochemical (Figures 1, 4, and 9) and histopathological results (Figure 10(c)).

This study suggests that the normal practice of UNCP consumption during malaria infection and treatment with A/L may have additional beneficial effects [17, 18, 59] in view of the fact that cocoa is also reported to have antimalarial properties. It is recommended that more studies be conducted to evaluate the possible synergistic antimalarial effect during concomitant administration of UNCP and A/L in addition to further investigating the mechanism of the hepatoprotective effect of UNCP. The findings from this study are consistent with previous studies that have shown that UNCP had reduced liver damage in mice infected with* Plasmodium berghei* [59], prevented alcohol-induced hepatic damage in rats [60], and shown hepatoprotective activity against carbon tetrachloride toxicity [61]. Further, it is highly possible that the hepatoprotective potential of UNCP could be due to both pharmacological properties of the macro- and microelements and its phytochemical composition. Regular consumption of UNCP has immense health benefits and its simultaneous administration with A/L during malaria treatment could be beneficial and should be encouraged. The daily administration 300 mg/kg, 900 mg/kg, and 1500 mg/kg bwt UNCP in these animals is equivalent to a daily amount of 4.54 g, 13.42 g, and 22.70 g of UNCP, respectively, in a 70 kg man according to Reagan-Shaw et al. [64].

## 5. Conclusion

Unsweetened natural cocoa powder has 38 macro- and microelements, increases nitric oxide levels, and has hepatoprotective potential during high dose A/L administration. The simultaneous consumption of UNCP and A/L is not likely to result in liver injury or dysfunction. Care must however be taken during high daily consumptions due to the high copper content.

## Figures and Tables

**Table 1:** Mean and standard deviation (SD) of measured elements (mg/4000 mg).

Element	Mean/SD mg/4000 mg
Macroelements	
Na	2.4666 ± 0.00
Mg	33.0133 ± 0.02
Al	14.0093 ± 0.01
Si	15.3880 ± 0.02
P	64.3866 ± 0.00
S	30.9120 ± 0.00
Cl	2.3616 ± 0.00
K	149.0667 ± 0.03
Ca	11.0146 ± 0.00
Ti	0.0232 ± 0.00
Mn	0.4093 ± 0.00
Fe	1.0309 ± 0.00

Microelements	
V	0.2320 ± 1.73
Cr	0.4200 ± 17.44
Co	0.0108 ± 0.10
Ni	0.0638 ± 1.16
Cu	0.2984 ± 1.71
Zn	0.4086 ± 0.74
Ga	0.0024 ± 0.00
As	0.0020 ± 0.00
Rb	0.1698 ± 0.49
Sr	0.1064 ± 0.20
Y	0.0016 ± 0.00
Zr	0.0125 ± 0.42
Nb	0.0070 ± 0.29
Mo	0.0044 ± 0.00
Sb	0.0043 ± 0.06
I	0.0133 ± 0.15
Cs	0.0232 ± 0.10
Ba	0.0620 ± 5.81
La	0.0480 ± 0.00
Ce	0.0849 ± 4.97
Hf	0.0148 ± 0.17
Ta	0.0213 ± 0.06
Pb	0.0036 ± 0.00
Bi	0.0024 ± 0.00
Th	0.0020 ± 0.00
U	0.0112 ± 0.10

## Abbreviations

FDA: Food and Drugs Authority
EDXRF: Energy dispersive X-ray
RDA: Recommended daily allowance
UNCP: Unsweetened natural cocoa powder
A/L: Artemether/lumefantrine
ALT: Alanine aminotransferase
AST: Aspartate aminotransferase
ALB: Albumin
ALP: Alkaline phosphatase
ANOVA: Analysis of variance
GAFCO: Ghana Agriculture Food Company
LD: Low dose
MD: Medium dose
HD: High dose
S-D: Sprague-Dawley
SDR: Sprague-Dawley rats
NeLP: Necrotic liver parenchyma
CCV: A highly congested and dilated central vein
LYM: Lymphocytic infiltration
DCV: Dilated central vein
UCV: Uncongested central veins.

## Additional Points

Availability of data and materials: Coartem sample and unsweetened natural cocoa powder are legally registered products in Ghana and samples deposited at the University of Ghana School of Pharmacy. Data of the above studies and the photomicrographs are available in the Department of Pharmacology and Toxicology, University of Ghana. The limitations of this study was the fact that animals infected with specific malaria parasites could have been used as a source of comparison and the fact that other species of animals could also be used to conduct this research. It is recommended that a separate study be conducted to investigate the effect of UNCP on parasitemia during A/L administration in malaria infected animals and specific mechanisms under which this protective effect occurs.
